# Determining Associations between Human Diseases and non-coding RNAs with Critical Roles in Network Control

**DOI:** 10.1038/srep14577

**Published:** 2015-10-13

**Authors:** Haruna Kagami, Tatsuya Akutsu, Shingo Maegawa, Hiroshi Hosokawa, Jose C. Nacher

**Affiliations:** 1 Department of Information Science, Faculty of Science, Toho University, Funabashi, 274-8510, Japan; 2 Bioinformatics Center, Institute for Chemical Research, Kyoto University, Uji 611-0011, Japan; 3 Department of Intelligence Science and Technology, Graduate School of Informatics, Kyoto University, Kyoto 606-8501, Japan

## Abstract

Deciphering the association between life molecules and human diseases is currently an important task in systems biology. Research over the past decade has unveiled that the human genome is almost entirely transcribed, producing a vast number of non-protein-coding RNAs (ncRNAs) with potential regulatory functions. More recent findings suggest that many diseases may not be exclusively linked to mutations in protein-coding genes. The combination of these arguments poses the question of whether ncRNAs that play a critical role in network control are also enriched with disease-associated ncRNAs. To address this question, we mapped the available annotated information of more than 350 human disorders to the largest collection of human ncRNA-protein interactions, which define a bipartite network of almost 93,000 interactions. Using a novel algorithmic-based controllability framework applied to the constructed bipartite network, we found that ncRNAs engaged in critical network control are also statistically linked to human disorders (P-value of *P* = 9.8 × 10^−109^). Taken together, these findings suggest that the addition of those genes that encode optimized subsets of ncRNAs engaged in critical control within the pool of candidate genes could aid disease gene prioritization studies.

RNA research has attracted increasing attention in recent years. Empirical evidence shows that most of the encoded information in the genomes of higher organisms, such as mammals, is transcribed into non-protein-coding RNAs (ncRNAs), which can be explained by the unbalanced complexity scale between prokaryotic and eukaryotic organisms^1^. Although these RNA molecules are not further translated into proteins, they can still play key biological functions in a cell. Numerous families of ncRNAs have been identified and classified, including rRNAs, tRNAs, snRNAs (small nuclear RNAs), snoRNAs (small nucleolar RNAs), miRNAs (micro RNAs) and many long ncRNAs. These molecules can be expressed in cells, but rather than coding a specific protein, they target and modify the expression of other biomolecules^2^. Whereas infrastructural RNAs (rRNAs and tRNAs) have been typically assigned to functions related to protein synthesis, small RNA molecules (snoRNAs, miRNAs and siRNAs) have shown the ability to perform multilevel regulatory tasks by altering protein expression levels^3^. For example, miRNAs can be involved in cell growth, stem cell functions, cell proliferation and embryonic development^4^ and have been found to target the genes with high transcriptional regulation complexity^5^. Moreover, numerous researchers have reported multiple associations between non-coding RNAs and complex diseases, including viral infections and oncogenesis^6^,^7^,^8^,^9^,^10^,^11^. There is evidence indicating that mutations and dysregulation of miRNAs may result in various diseases^12^,^13^,^14^,^15^. These findings have recently led to the development of promising miRNA-targeting therapeutics^16^,^17^.

Although the sequence information of ncRNAs is available, until recently, the interactions between specific ncRNAs and other protein molecules have not been collected and classified in large numbers. A recent major update of the NPInter database v2.0^18^, which includes more than 200,000 interactions (only 700 were reported in v1.0^19^) of ncRNAs with other bio-molecules from 18 organisms, including humans, with almost 93,000 interactions, offers a promising opportunity to investigate the large-scale structure of the ncRNA-protein interaction network and to determine to what extent each ncRNA is engaged in network regulation and control using the latest advances in network controllability.

Recent developments in network science have provided a variety of methods to investigate the controllability feature in large-scale networks in directed^20^ and undirected unipartite networks^21^,^22^. Developments in structural controllability in bipartite networks using the Minimum Dominating Set (MDS) have also provided the necessary methodology to formalize its study^23^. Molnár *et al.* exhaustively investigated the variations of the MDS with respect to various types of network structures using a greedy algorithm^24^. On the biological side, the application of domination techniques is also promising. A hitting set formulation, which is equivalent to set cover in bipartite networks, has been used to uncover 14 anticancer drug combinations using data from 60 tumor derived cell lines^25^. Domination analysis of biological networks has shown a statistically significant enrichment of topological central genes in aging, cancer, infectious diseases, and signaling pathways^26^. Wuchty investigated the controllability in protein interaction (PPI) networks (a unipartite network) and discovered that an optimized subset of proteins (MDS) was enriched with essential, cancer-related, and virus-targeted genes^27^. Moreover, these identified proteins are highly involved in regulatory functions, showing high enrichment in transcription factors and protein kinases, and participate in regulatory links, phosphorylation events, and genetic interactions. However, previous biological analyses were performed using only one of the multiple MDS configurations. Because the computation of the MDS does not lead to a unique set of controllers, it is possible to perform a more precise network control analysis and to distinguish between several control categories, such as critical, intermittent and redundant^20^. Hence, the controllers can be classified into three classes depending on how they are engaged in network control. Moreover, PPI is a unipartite network, and in this work, we focus on a bipartite network (ncRNA-protein interactions), which increases the analysis complexity.

Here, we present a novel computational procedure to calculate the fraction of critical, intermittent and redundant nodes in a bipartite network, which extends previous computational methodology specifically derived for unipartite networks^20^. By using the proposed algorithmic framework implemented on bipartite networks and human ncRNA disease associations collected from the HMDD^29^ and OMiR^30^ databases, we can identify an optimized subset of ncRNA controllers that exhibits a statistically significant enrichment with human disorder classes, unveiling a novel link between ncRNA molecules that are highly involved in critical network control and specific diseases.

## Methods

### ncRNA-protein interactions and diseases associations datasets

Interactions between non-coding RNA and proteins were retrieved from the NPInter database v2.0^18^. This database consists of experimentally reported interactions between non-coding RNAs and other biomolecules, including proteins, RNAs and genomic DNAs. We selected the human organism and extracted the molecular interactions corresponding to ncRNAs and proteins, which led to a subset of 3,894 ncRNAs, 5,783 proteins and 92,998 interactions. We used the HMDD database (version 2)^29^ and the OMiR database^30^ for human microRNA and disease associations and for associations between ncRNAs and “orphan” Mendelian diseases, respectively (see SI for details).

### Determining the minimum set of critical controllers in the ncRNA-protein network

We analyzed the controllability features of the non-coding RNA-protein bipartite network. A bipartite graph *G*(*V*_*T*_*,V*_*B*_; *E*) consists of a set of top nodes *V*_*T*_ and a set of bottom nodes *V*_*B*_. A set of edges connects both sets of nodes (*E* ⊆ *V*_*T*_ × *V*_*B*_). The set of edges represents directions from *V*_*T*_ to *V*_*B*_. A set *S* ⊆ *V*_*T*_ of nodes in the graph G is a *dominating set* if for all nodes *w* ∈ *V*_*B*_, there exists a node *v* ∈ *V*_*T*_ such that (*v, w*) ∈ *E*. This dominant set (DS) of nodes plays the role of the set of driver nodes^23^. In our problem, the set of top nodes corresponds to ncRNA molecules and the set of bottom nodes to proteins. Hence, the set of controllers corresponds to a subset of *V*_*T*_ (ncRNAs). The minimum number of ncRNA controllers can be identified by calculating the dominating set of minimum cardinality i.e. Minimum Dominating Set (MDS)^23^. The optimal solution for the MDS problem in bipartite network is obtained by using Integer Linear Programming (ILP) (see Eq. 1 in the SI). Although it is an NP-hard problem, surprisingly, the exact solution can be obtained for large networks up to more than 10^5^ nodes within a few seconds^23^ (see the SI for details). Because the computation of the MDS does not lead to a unique set of controllers, we can classify the nodes depending on their network control roles. The novel algorithm that uses an MDS-based method to determine the minimum set of critical and redundant controllers for a bipartite network is presented in the SI. The set of critical nodes represents those nodes that belong to every MDS configuration and therefore always play a role in network control. The set of redundant nodes denotes those nodes that never appear in any MDS configuration and therefore are never engaged in controllability roles. Finally, those nodes that appear in some MDS but not in all MDS configurations are called intermittent nodes. The fraction of intermittent nodes *n*_*i*_ can be computed from the fractions of critical *n*_*c*_ and redundant *n*_*r*_ nodes as follows: *n*_*i*_ = 1 − *n*_*c*_ − *n*_*r*_. We also performed a mathematical analysis to theoretically estimate the fraction of critical nodes which is shown in the SI. Enrichment calculation is done as in^27^ (see SI for details).

## Results

### Network structure of the ncRNA-protein interaction network

**Figure 1:**
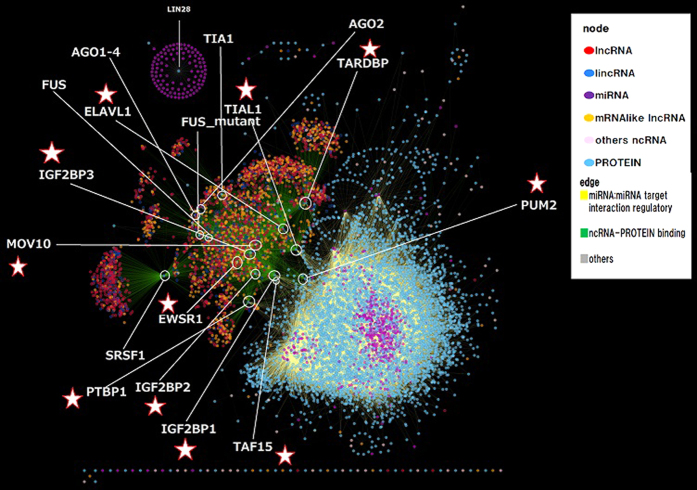
Visualization of the bipartite ncRNA-protein interaction network. Color legend denotes each main ncRNA class. The largest fraction of the interactions corresponds to the miRNA target interaction and regulatory class, which includes 85,355 (yellow) edges. A second large group is composed of 8,162 interactions (green) edges and is associated with the ncRNA-protein binding class. Proteins (nodes in blue) with the highest number of interactions are highlighted. The star symbol indicates that the protein plays a ‘bridge’ role.

Using the NPInter v2.0 database, we extracted the molecular interactions corresponding to ncRNAs and proteins in humans. A visual representation of the entire network is shown in Fig. 1. The NPInter database includes a number of non-coding RNAs classes. A total of 32 classes were involved in the construction of the ncRNA-protein interaction network for humans. The color legend in Fig. 1 denotes each main ncRNA class, and Table 1 shows the statistics of each class. The miRNA class is the third largest class, including 796 molecules, after the lncRNAs related classes, and it exhibits the highest average degree. One possible reason for these unbalanced degree values is that miRNAs have been studied in more detail than newly discovered ncRNA classes, and their interactions and disease associations have been studied more systematically. Indeed, Fig. 1 also illustrates that the largest fraction of the interactions corresponds to miRNAs, namely the miRNA target interaction and regulatory class, which includes 85,355 (yellow) edges. A second large group is composed of 8,162 interaction (green) edges and is associated with the ncRNA-protein binding class. Other small groups of interaction classes, such as expression correlation, with only 27 interactions, are denoted in grey in Fig. 1.

**Figure 2:**
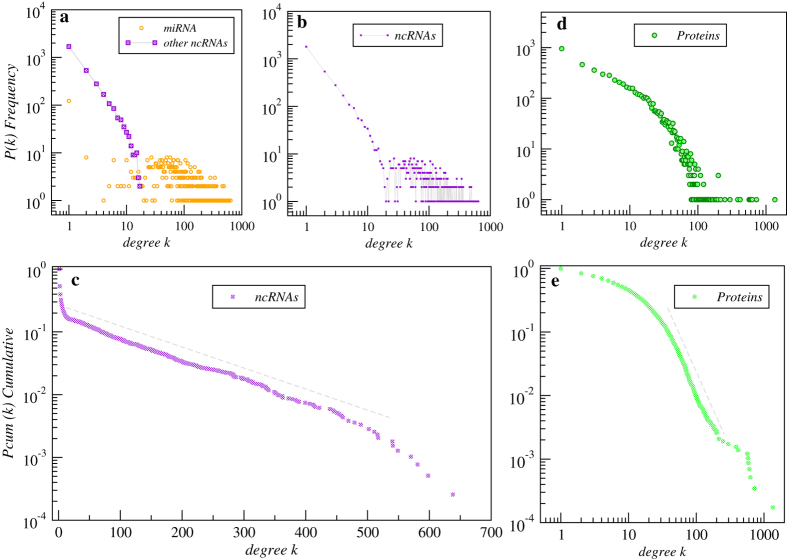
Degree and cumulative distributions for ncRNAs and proteins. (**a**) Decomposition of the out-degree distribution for miRNAs (purple) and the combination of the rest of the ncRNA classes (orange). (**b**) Degree distribution for all combined ncRNA classes (**c**) Cumulative degree distribution for the ncRNAs on a log-linear scale. (**d**) In-degree distribution for proteins. (**e**) Cumulative degree distribution for proteins on a log-log scale. Data for the fitting analysis is shown and discussed in the main text.

To analyze the global structure of the bipartite ncRNA-protein interaction network, we used the degree distribution. The results shown in Fig. 2(d,e) indicate that the protein degree distribution has a range of several decades, compatible with a power-law distribution from *k*_min_ = 41.1 ± 5.2 and characterized by a degree exponent *γ* = 3.28 ± 0.15. The degree distribution for the ncRNAs shown in Fig. 2(a–c) suggests a more complicated picture. As shown in Table 1, the component related to miRNAs is highly connected, and its degree distribution analysis reveals that it tends to decay exponentially. In contrast, the rest of the ncRNAs are less densely connected to proteins (see Table 1), and their degree distribution tends to follow a power-law decay for low degrees. The asymmetric degree distributions of these two large components of the same network are highlighted in Fig. 2(a,b). The explained tendency is more evident when the cumulative degree distribution *P*( > *k*) is plotted on a log-linear scale, showing that, only from high degrees above *k* > *10,* the distribution follows an exponential decay of the form *e*^*−λ.k*^ with *λ* = 0.008 ± 0.001.

Three main findings can be derived from this analysis. First, there is a nonzero probability to find highly connected proteins interacting with many ncRNAs (see Fig. 2(d,e)). Second, a large fraction of ncRNAs interact with a similar number of proteins. Third, the unveiled structure of the ncRNA-protein interaction network displays an uncommon topology, characterized by two connected but drastically different sub-networks, one led by miRNA and the other consisting of the rest of the ncRNAs, mainly dominated by long ncRNAs (lncRNAs). By only removing 11 proteins (highlighted by a star symbol in Fig. 1), both large sub-networks become topologically disconnected. The biological functionalities of these ncRNA-bridges related proteins are shown in Table 2. Because the tendency of the protein degree distribution is a power-law, there should be a small set of highly connected proteins. The degree of each hub is also shown in Table 2. Most of the highly connected proteins are related to lncRNA, and low degree proteins tend to be associated with the miRNA component.

### A small number of ncRNAs control the entire network

**Figure 3:**
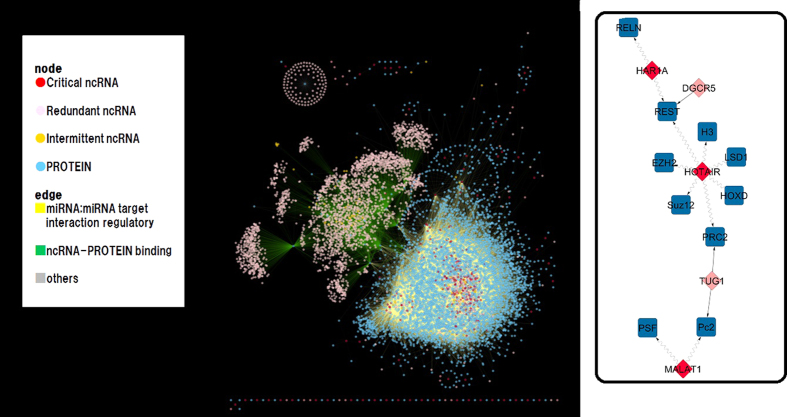
Controllability in the ncRNA-protein interaction network. (Left) Visualization of the distribution of critical, intermittent and redundant nodes in the entire network. Color indicates node control functionality and molecule type as shown in the legend. (Right) Highlight of a network subgraph that illustrates an example of critical network control. Network control is indicated by the directed waved lines. Ten proteins are controlled by only three ncRNAs playing a critical role. The remaining two ncRNAs are redundant.

The computation of an MDS in the bipartite ncRNA-protein interaction network allows us to identify the minimum number of controllers needed to achieve full network control (see Fig. 3). The total number of ncRNAs collected in our study is 3,894. Among them, only 371 are needed to simultaneously control 5,783 proteins using the MDS approach, which represents only 9.5% of the ncRNAs (see Table 1 for the distribution of the MDS size in the ncRNA classes). More importantly, we also applied the algorithm to compute the number of critical and redundant ncRNAs under the MDS framework. The results show that 335 and 3,419 nodes play critical and redundant roles, respectively (see Table 1 for the distribution of the size of the critical set in ncRNA classes). Most of the nodes involved in critical control belong to the miRNA class. A small fraction of ncRNAs, 140, is engaged in intermittent network control, representing 3.5% of the total ncRNAs. By combining the critical and intermittent nodes, the total number of ncRNAs engaged in network control only represents 12.2%. In this bipartite network, the numbers of MDS and critical nodes are relatively similar. Unipartite networks, in contrast, tend to show different numbers for MDS and the critical set^20^. However, from a functional viewpoint, the critical nodes are more important because they are always engaged in network control regardless of the arbitrarily selected MDS configuration. This essential distinction between MDS and critical roles is missing in ref. 27. Figure 3 and Table 1 show that a large number of nodes involved in critical control belong to the miRNA class.

 We also performed a simple theoretical analysis to estimate the fraction of critical nodes (See SI for details). Table 3 shows the value of Eq. 2 shown in SI and the actual fraction of critical nodes in the ncRNA-protein interaction network for each out-degree *k*. Eq. 2 gives good estimates for low out-degree nodes, whereas it does not for larger out-degree nodes, due to degree correlations or heterogeneity.

### Enrichment of critical and redundant nodes in ncRNA-protein interactions

**Figure 4:**
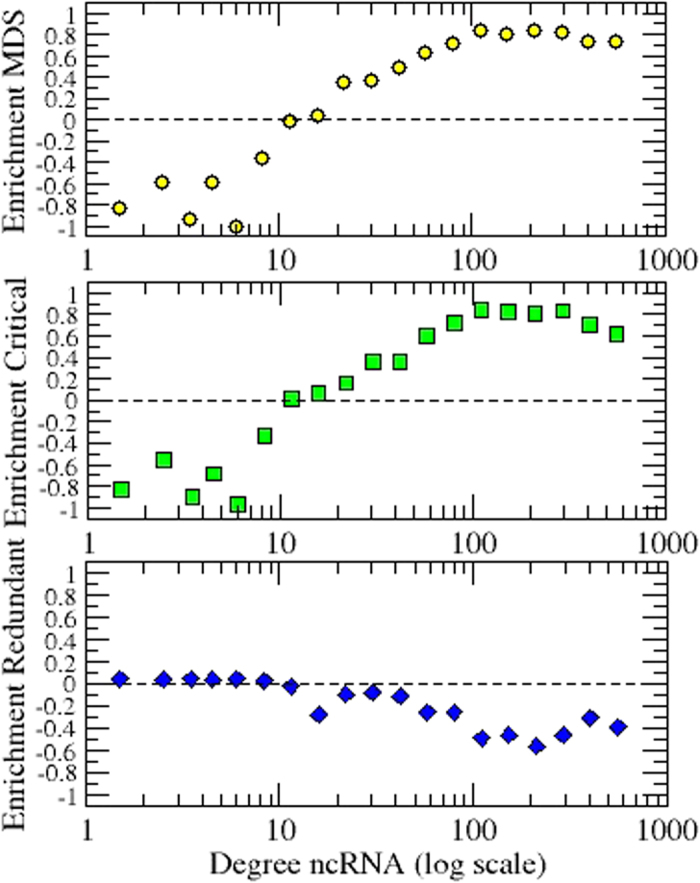
Degree enrichment of the MDS, critical and redundant sets. Enrichment results that show the statistical proportion of ncRNAs engaged in a given set *S*, such as the MDS, critical or redundant, according to their degrees.

To evaluate the enrichment of controllability features as a function of the connectivity of the ncRNAs, we classified ncRNAs according to their degree in bins of logarithmic increasing size. In each bin class, we computed the enrichment as defined in the Methods section. Figure 4 shows that the MDS and Critical set are clearly enriched with ncRNAs that have more than 10 outgoing links. Conversely, the redundant set is depleted with ncRNAs that have more than 10 interactions. For each ncRNA class, we also investigated the enrichment levels (Fig. S1). Although most of the classes are populated by few ncRNAs, some classes contain many molecules. Among the latter, miRNAs show the highest enrichment. The largest classes, which are related to long non-coding RNAs (lncRNAs), show a depletion in controllability roles. It is worth mentioning that the lncRNAs^31^,^32^ and the large intergenetic non-coding RNAs (lincRNAs)^33^,^34^ are novel heterogeneous classes that are rapidly emerging in literature and being progressively associated to a myriad of biological functions. However, because they have been discovered much more recently than miRNAs, they not only remain less-well understood but also their regulatory interactions are less exhaustively catalogued, which may explain their low average degree shown in Fig. 1 and Table 1.

### Enrichment of critical and redundant nodes in ncRNA disease associations

Annotations regarding disease associations from the HMDD database resources were mapped to the ncRNAs obtained from the NPInter database. We then classified the ncRNAs into two groups based on whether they have a disease or non-disease association. Another classification was performed for ncRNAs based on whether they belong to the MDS and the critical set of nodes. Among all possible MDS configurations, we selected one and classified the ncRNAs as shown in Table S1 as a contingency table. The total number of different diseases included in the database is 367. Using the results shown in Table S1, we applied Fisher’s exact test to determine whether the MDS of ncRNAs is significantly enriched with disease associations. The result of the test was a two-tailed exact P-value with a strong signal (*P* = 1.0 × 10^−124^). Therefore, the associations between disease and ncRNAs that belong to the MDS are statistically significant.

Because the MDS is not unique, we focus on those critical nodes that are always engaged in network control. The results for the critical set of ncRNAs are shown in Table S1. Applying Fisher’s exact test, we found that diseases were significantly enriched in the critical set of ncRNAs with a two-tailed exact P-value of *P* = 9.8 × 10^−109^. A histogram with the number of ncRNAs that play a critical role in network control and associated to each disease is shown in Fig. S2. Out of all diseases, the histogram only shows data for the top 28 diseases with the highest number of ncRNAs engaged in critical network control and associated with the disease. The histogram is dominated by hepatocellular carcinoma and stomach, breast and colorectal neoplasms.

**Figure 5:**
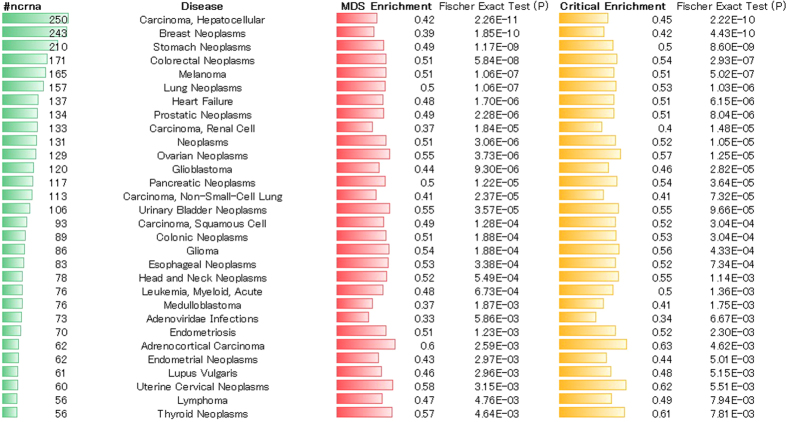
Disease enrichment of the MDS and critical set. The figure shows the results for the top 30 diseases with the highest number of ncRNA associations. Next to the enrichment scores for the MDS and critical set, the two-tailed P-values for the Fisher’s exact test are displayed. A full list with all diseases is shown in Table S2. The result demonstrates that for each disease, there is a significant enrichment in both the MDS and critical set. For the statistical significance tests, a two tailed p-value of more (less) than 0.05 rejects (accepts) the hypothesis of disease association with the MDS and critical set.

Next, we investigated the enrichment of MDS and the critical set of ncRNAs for each particular disease. The results for the top 30 diseases with highest number of ncRNA associations are shown in Fig. 5. Next to the enrichment scores, the two-tailed P-values for the Fisher’s exact test are displayed. A full list with all diseases is shown in Table S2. The result demonstrates that for each disease, there is a significant enrichment in both MDS and the critical set. When only diseases that passed the Fisher’s exact test are considered, the enrichment of the critical set is, in most cases, higher than that of the MDS (Fig. S3), which reinforces the importance in network control. Moreover, the enrichment function does not depend strongly on the size of the MDS or the critical set involved in the disease and, on average, is distributed at approximately 0.5 (Fig. S4). The results of the analysis using a different data repository, such as the lncRNADisease database, are shown in the SI.

Finally, we investigated the ncRNA-disease associations reported in the OMiR dataset. The total number of diseases included in the database is 79. By applying the Fisher’s exact test to the data shown in Table S4, we found that the MDS of the ncRNAs is enriched with orphan diseases (*P* = 6.0 × 10^−43^). We hypothesized that the critical set of the ncRNAs is also enriched with disease associations. The Fisher’s exact test result showed a statistically significant association between the critical set of the ncRNAs and the set of “orphan” Mendelian diseases (*P* = 3.3 × 10^−40^) (see SI for details).

A novel emerging class of ncRNAs consists of long non-coding RNAs (lncRNAs), which are being widely identified in large numbers within mammals and associated to important tasks in many different cellular processes such as regulating gene expression at different stages, from epigenetic to transcriptional and post-transcriptional^31^,^32^. Recently, Liao *et al.* predicted the functions of lncRNAs based on coding-non-coding gene co-expression network method^35^. Our approach does not make use of the gene expression information. In contrast, the MDS methodology uses the reported experimental biological interactions to identify a mathematically optimized set of controllers operating as a critical role. Out of 1507 lncRNAs analysed in our work, only six lncRNAs belong to the identified MDS and five out of the six were classified as critical controllers. The lncRNA that belongs to the MDS but not to the critical set is the RPI001_2629 that interacts with protein O43251 (also known as RNA binding protein fox-1 homolog 2), and is encoded in the gene RBFOX2. This interaction is classified as ncRNA-protein binding interaction class according to the NPInter annotation. In contrast, all of five critical lncRNAs identified in our study have been reported as controllers in literature and classified as regulatory interaction classes in annotations from NPInter database. They were not, however, identified as regulators by using the co-expression network model whose predictions are provided in NONCODE database^35^. The CBR3-AS1, synonym of the PlncRNA-1, is a recently discovered prostate cancer-up-regulated long noncoding RNA, which modulates apoptosis and proliferation through reciprocal regulation of androgen receptor^36^. The LINC00312, also known as ERR (estrogen receptor repressor)-10 has been reported as a repressor in transcriptional signaling activation of estrogen receptor-alpha^37^. Next, the long noncoding RNA RNCR2 (retinal non-coding RNA 2), synonym of MIAT (myocardial infarction associated transcript), plays a critical role in regulating mammalian retinal cell fate specification^38^. The NCRUPAR-PAR1 (ncR-uPAR upregulated PAR-1) is a noncoding RNA that regulates human protease-activated receptor-1 gene during embryogenesis^39^. Finally, PVT-1 has been extensively investigated and reported as a regulator of the c-Myc gene transcription over a long distance^40^,^41^. Note also that all of these five lncRNAs identified as critical controllers by our approach are also associated to regulatory classes in NPInter v2.0 database. We believe that once lncRNAs and lincRNAs protein interactions are systematically collected and widely classified, a more detailed study of their controllability features could be very interesting, which may lead to identification of a larger number of controllers associated to critical roles.

The transcripts hsa-miR-20a, -20b, -93, -17, -106a, and -16b are known as the miR-17 family. The miR-17 family is related to pivotal biological processes, such as cell cycle regulation, cell death, and embryonic development^42^. All precursors give rise to microRNA with the sequence “AAAGUG” as the “seed” sequence. The miR-17 family miRNAs were first identified as an oncogene^43^,^44^. In fact, the miR-17 family microRNAs are overexpressed in human B-cell lymphoma and chronic lymphocytic leukemia^42^,^43^. In addition, members of the miR-17 family suppress the amyloid precursor protein APP directly *in vitro*^45^,^46^. In Alzheimer’s disease, downregulation of miR-106b has been reported in the patients’ brains. The multiple roles of the miR-17 family may be the reason why the miR-17 family was detected as important hubs in ncRNAs.

ELAVL1, IGF2BP2, IGF2BP3, and PUM2 were identified as hub proteins. These proteins are regulators of the translocation and/or translation of target mRNAs. ELAVL1 physically interacts with the AU-rich element in the 3′UTR of target RNAs and stabilizes the bound mRNAs, resulting in activation of protein synthesis^47^,^48^. In fact, ELAVL1 stabilizes a variety of target mRNAs, especially mRNAs related to cancer and inflammation^49^. IGF2BP2 and 3 also bind target mRNAs and regulate mRNA localization and translation of the target mRNAs^50^. In contrast, PUM2 has been identified as a repressor of translation from the target mRNAs^51^. PUM2 may have important roles in germ cell development because PUM2 physically interacts with DAZ (Deleted in azoospermia) protein, which is essential for germ cell development^52^. Thus, our method revealed relationships among important microRNAs and RNA-binding proteins involved in several biological processes and human diseases.

Some miRNAs in the miR-17 family are critical (or in the MDS) and others are not, which suggests that their functions are slightly different or that currently available data are insufficient. Although whether a node is critical (or in the MDS) is a good measure to evaluate the importance of the node, it is a binary measure and may not be robust for certain types of small changes of network topology. Future work should extend these measures to quantitative ones.

## Conclusions

The combination of disease annotation information with bipartite ncRNA-protein interaction network allowed us to investigate the statistical association between ncRNA controllers and human disorders and eventually led to our main finding. This question was analyzed using polygenic diseases, which included cardiovascular and cancer disorder clusters among others and ‘orphan’ Mendelian diseases, of which the disorders are typically less studied and are thought to be single-gene diseases^53^. The association between the identified optimized critical set of control nodes and both groups of diseases was statistically significant. This means that those ncRNAs that are always engaged in critical network control are also likely to be responsible for human disorders, which is our main result. These results also significantly extended those by Wuchty^27^ who only considered the minimum dominating sets in protein interaction network (a unipartite network). MDS is useful methodology for analyzing biological networks having bipartite structures, but as described above critical nodes are more important than MDS nodes. This work proposed the algorithmic procedure to identify critical nodes in complex bipartite networks.

Disease-associated genes are usually identified using linkage mapping or genome-wide association studies. More recently, however, disease module and diffusion-based methods computed on interactome networks have also contributed toward identifying disease genes^54^,^55^,^56^. In any methodology, however, the procedure requires a set of candidate genes, excluding ncRNAs, which represent the highest proportion of the human genome, to be pre-selected for the analysis. Our findings suggest that the genes that encode the small, optimized subset of non-coding RNAs enriched in human disorders and that play a critical role in network control could also be added to the pool of candidate genes to aid and improve disease gene prioritization.

## Additional Information

**How to cite this article**: Kagami, H. *et al.* Determining Associations between Human Diseases and non-coding RNAs with Critical Roles in Network Control. *Sci. Rep.* **5**, 14577; doi: 10.1038/srep14577 (2015).

## Supplementary Material

Supplementary Information

## Figures and Tables

**Table 1:** Statistics of the ncRNA and optimized subsets of control.

ncRNA Class	# ncRNAs	# MDS	# Critical	Av. Degree <k>
BC200 RNA	1	1	1	8
H19 RNA	1	1	1	9
MESTIT1 RNA	1	1	1	1
RNase P RNA	1	1	1	7
SRP RNA	1	1	1	8
telomerase RNA	1	1	1	8
TSU RNA	1	1	1	2
vault RNA	1	1	1	2
XIST RNA	1	1	1	6
KvLQT1-AS RNA	1	1	0	1
snRNA	9	6	6	7
IPW RNA	2	1	1	1.5
7SK RNA	2	1	1	8.5
Unclassified	19	8	8	1.8
miRNA	796	316	283	107.2
Y RNA	4	1	1	3.7
snoRNA	7	1	1	3.5
scaRNA	8	1	1	3.1
mRNAlike lncRNA	1088	19	18	2.6
lncRNA	1507	6	5	2.2
lincRNA	426	1	1	2.6
bic RNA	1	0	0	1
DISC2 RNA	1	0	0	1
GNAS1-as RNA	1	0	0	1
guide_RNA	2	0	0	4
H_ACA_box_snoRNA	1	0	0	7
NCRMS RNA	1	0	0	1
RNase MRP RNA	2	0	0	7.5
scAlu RNA	1	0	0	3.1
SRP_7SL RNA	3	0	0	1.3
UBE3A antisense RNA	2	0	0	2
aHIF RNA	1	0	0	1

The number of ncRNAs, MDS size, critical set size and the average degree for each ncRNA class is displayed.

**Table 2:** Annotated information and functionality of proteins with the highest degree.

Gene ID	Degree	Feature	Uniprot ID	Protein functional description
ELAVL1	1307	Bridge	Q15717	Binds avidly to the AU-rich element in FOS and IL3/interleukin-3 mRNAs.
IGF2BP3	713	Bridge	O00425	RNA-binding protein that act as a regulator of mRNA translation and stability.
SRSF1	629		Q07955	Plays a role in preventing exon skipping, ensuring the accuracy of splicing and regulating alternative splicing.
IGF2BP2	552	Bridge	Q9Y6M1	Binds to the 5′-UTR of the insulin-like growth factor 2 (IGF2) mRNAs.
TARDBP	540	Bridge	Q13148	DNA and RNA-binding protein which regulates transcription and splicing.
TIAL1	527	Bridge	Q01085	RNA-binding protein. Possesses nucleolytic activity against cytotoxic lymphocyte target cells.
IGF2BP1	522	Bridge	Q9NZI8	RNA-binding factor that affects mRNA nuclear export, localization, stability and translation.
FUS	413		P35637	Binds both single-stranded and double-stranded DNA.
PTBP1	327	Bridge	P26599	Plays a role in pre-mRNA splicing and in the regulation of alternative splicing events.
TIA1	296		P31483	Involved in alternative pre-RNA splicing and regulation of mRNA translation by binding to AU-rich elements (AREs).
EWSR1	246	Bridge	Q01844	Might normally function as a transcriptional repressor.
FUS_mutant	216		P35637	Binds both single-stranded and double-stranded DNA.
AGO1-4	199		Q9UL18;	Required for RNA-mediated gene silencing (RNAi).
AGO2	180		Q9UKV8	Required for RNA-mediated gene silencing (RNAi) by the RNA-induced silencing complex (RISC).
TAF15	173	Bridge	Q92804	RNA and ssDNA-binding protein that may play specific roles during transcription initiation at distinct promoters.
MOV10	169	Bridge	Q9HCE1	Probable RNA helicase. Required for RNA-mediated gene silencing by the RNA-induced silencing complex (RISC).
PUM2	132	Bridge	Q8TB72	Sequence-specific RNA-binding protein that regulates translation and mRNA stability.
TNRC6A-C	92		Q8NDV7;	Plays a role in RNA-mediated gene silencing by both micro-RNAs (miRNAs) and short interfering RNAs (siRNAs).
QKI	85	Bridge	Q96PU8	RNA-binding protein that plays a central role in myelinization.

The set of proteins with ‘bridge’ features indicate that its removal leads to a disconnected work, as explained in the text.

**Table 3:** Comparison of the theoretical predictions and experimental data results.

Binned degree	Theory	Exp. data
1	0.010	0.012
2	0.020	0.024
4	0.040	0.013
8	0.079	0.029
16	0.152	0.089
32	0.281	0.174
64	0.483	0.256
128	0.732	0.513
256	0.928	0.570
512	0.994	0.511
1024	0.999	0.333

The theoretical analysis derives a simple mathematical expression (Eq. 2 in SI) that can estimate the fraction of critical nodes in bipartite networks.
