# Supplementary material for: Determining Associations between Human Diseases and non-coding RNAs with Critical Roles in Network Control
Source: Sci Rep. 2015 Oct 13;5:14577. doi: 10.1038/srep14577 (PMC4602215; doi:10.1038/srep14577)
Supplement: Supplementary Information [file srep14577-s1.doc]

**Supplementary Information**

**Determining Associations between**

**Human Diseases and non-coding RNAs**

**with Critical Roles in Network Control**

*Haruna Kagami, Tatsuya Akutsu, Shingo Maegawa,*

*Hiroshi Hosokawa and Jose C. Nacher*

1. **METHODS**

## Human microRNA and disease associations

We used the human miRNA-associated database (HMDD), which includes miRNA annotations, disease names and evidence of dysfunction. The latest updated dataset Version 2.0, available in 2013 and used in our research, includes associations for 367 human disorders [1].

## OMiR: Associations between ncRNAs and “orphan” Mendelian diseases

In general, less common human disorders have been less investigated. Although 1,822 protein-encoding genes in humans are estimated to be related to monogenic disease, the annotation of more than 1,500 of these genes remains unknown. These single-gene diseases are referred to as “orphan” diseases. The OMiR database is based on an algorithm that computes and classifies the relations between miRNA and genetic diseases [2] and includes the annotations of small ncRNAs and their associations to rare diseases via OMIM annotations [3]. A total of 79 human disorders associated with ncRNAs were collected from this database.

**Determining the set controllers in ncRNA-protein network**

Here we analyze the controllability features of the non-coding RNA-protein bipartite network. A bipartite graph consists of a set of top nodes *V_T_* and a set of bottom nodes *V_B_*. A set of edges connects both sets of nodes . The set of edges represents directions from *V_T_* to *V_B._*  In our problem, the set of top nodes corresponds to ncRNA molecules and the set of bottom nodes to proteins. Hence, the set of controllers corresponds to a subset of *V_T_* (ncRNA). A set $S\subseteq V_{T}$ of nodes in the graph G is a *dominating set* if for all nodes $w\in V_{B}$ , there exists a node $v\in V_{T}$such that $(v,w)\in E$. This dominant set (DS) of nodes plays the role of the set of driver nodes [4]. In our problem, the set of top nodes corresponds to ncRNA molecules and the set of bottom nodes to proteins. Hence, the set of controllers corresponds to a subset of *V_T_* (ncRNA). The minimum number of ncRNA controllers can be identified by calculating the dominating set of minimum cardinality i.e. Minimum Dominating Set (MDS).

Note that an MDS of a bipartite network also corresponds to a minimum set cover. The computation of the MDS in graphs is an NP-hard problem [5-7]. The MDS has found applications in computer engineering and mobile systems therefore procedures for its computations have been extensively developed [8-9]. In particular, approximation algorithms have been developed to obtain approximate solutions to optimization problems for very large networks [5, 8]. However, the optimal solution for the MDS problem in bipartite networks can be computed in very large networks up to more than 10^5^ nodes within a few seconds [4]. Therefore, in this work, instead of using a greedy algorithm, the optimal and exact solution for the computation of the MDS in bipartite network is obtained by formalizing the problem as an Integer Linear Programming (ILP) expression as follows:

minimize

subject to

(Eq. 1)

Given an adjacency matrix corresponding to a bipartite network as input data, a C program creates an ILP instance given by Eq. 1. Then, the optimal solution is calculated using this ILP instance by means of the ‘glpsol’ solver. Hence, an MDS for a given bipartite network is obtained by the set

**New algorithm to Compute Critical and Redundant ncRNA molecules in a bipartite network**

As we have shown, not all of the MDS consists of the same nodes. Therefore, although the size the MDS is the same, the MDS may be composed of different ncRNA molecules. We can classify the nodes into critical, intermittent, and redundant nodes. The set of critical nodes represents those nodes that belong to every MDS configuration and therefore always play a role in network control. The set of redundant nodes denotes those nodes that never appear in any MDS configuration and therefore are never engaged in controllability roles. Finally, those nodes that appear in some MDS but not in all MDS configurations are called intermittent nodes. The fraction of intermittent nodes $n_{i}$ can be computed from the fractions of critical $n_{c}$and redundant $n_{r}$ nodes as follows: $n_{i}=1-n_{c}-n_{r}$.Of particular interest are the critical nodes because they are always members of all possible MDS and are always engaged in controlling a network. The identification of the critical nodes in controlling a bipartite network is as follows:

1. Using an ILP as defined in Eq. 1, we identify an MDS of size *M* for a bipartite network .
2. Let *B_MDS_* be an empty set.

Then, and we repeat steps 3-6.

1. By inserting a new constraint of into the instance shown by Eq. 1, a new ILP instance *I_v_* is defined.
2. Compute a solution of *I_v_* and define .
3. If or |, then we set
4. Return *B_MDS._*

Then, the redundant nodes can also be identified in a similar way. In contrast to the critical nodes, the redundant nodes are never members of an MDS set. Therefore, they are never engaged in control operations.

1. Using an ILP as defined in Eq. 1, we identify an MDS of size *M* for a bipartite network .
2. Let *D_MDS_* be an empty set.

Then, and we repeat steps 3-6.

1. By inserting a new constraint of into the instance shown by Eq. 1, a new ILP instance *I_v_* is defined.
2. Compute a solution of *I_v_* and define .
3. If or |, then we set
4. Return *D_MDS._*

In this work, both critical and redundant procedures were implemented using C programs. For each instance, the program called glpsol was applied to solve the ILP.

The fraction of nodes engaged in intermittent network control can be easily obtained because the sum of the fractions of the critical, redundant and intermittent nodes is 1. Intermittent and critical nodes are the only subsets of nodes that can be engaged in network control. The procedures are similar to those of the computation of the MDS in unipartite networks [10]. However, the algorithm has been adapted to bipartite networks, and the main difference comes from the fact that the ILP for MDS in bipartite networks is formalized in a different way, as shown in Eq. 1. Therefore, it allows, for the first time, to investigate the critical and redundant nodes in real-world bipartite networks, such as the ncRNA-protein network.

**Enrichment calculation**

The enriched ncRNAs in a given disease *D* that appear in a set such as MDS or critical set were processed as follows. First, we calculated the fraction of the number of nodes (ncRNAs) associated with a disease *D* ($N_{D}$) in the entire network of size *N* as $f_{D}=N_{D}/N$. Then, the fraction of the number of ncRNAs associated with a disease *D* that appeared in an MDS or critical set ($N_{D}^{S}$) of size $N^{S}$is calculated as $f_{D}^{S}=N_{D}^{S}/N^{S}$. Then, the enrichment of ncRNAs in a disease *D* for an MDS or critical set *S* is computed as $E_{D}^{S}=\log(f_{D}^{S}/f_{D})$.

To measure the statistical proportion of ncRNAs engaged in a given set *S* (such as the MDS, critical or redundant) according to their degrees, we used the following procedure, which was also used in Ref. [11]. First, the ncRNAs were classified according to their degree in logarithmic bins of increasing size. For each bin class *i,* we computed the frequency of ncRNAs with degree *k* as: . Similarly, we computed the frequency of ncRNAs with degree *k* that also appeared in a given set *S* as:

. Then, the enrichment of ncRNAs with degree *k* that appear in the set *S* (MDS, critical or redundant) in bin *i* was computed as Values of indicate the enrichment of degree *k* for the MDS, critical and redundant set, respectively. Negative values indicate depletion of degree *k*.

**Statistical significance tests**

The exact two-tailed p-value for the enrichment of the MDS and critical set of ncRNAs in human diseases was calculated using Fisher’s exact test. The statistical significance tests for the enrichment of MDS and critical in each disease were computed using one sample proportion test using the binomial exact test. The complete results for all the diseases are shown in Table S2, which includes the exact two-tailed Fisher p-value.

**Theoretical analysis**

We performed a simple theoretical analysis to estimate the fraction of critical nodes. In the following, in-degree (resp., out-degree) of a node means the number of incoming (resp., out-going) edges. The following proposition is a simple but important observation.

**Proposition 1.** If *v* in *V_T_* has at least one neighbor node *u* of in-degree 1 in *V_B_*, *v* is a critical node.

One neighboring node is enough in this proposition, whereas two neighboring nodes are needed for unipartite networks (Proposition 2.2 in Nacher and Akutsu [10]). Using this proposition, we estimate the fraction of critical nodes within out-degree *k* nodes where the proposition only suggests a lower bound.

Let *n*_1_ and *m* denote the number of in-degree 1 nodes in *V_B_* and the number of edges in , respectively. If we randomly choose one edge from *E*, the probability that its endpoint in *V_B_* is an in-degree 1 node is *p*_1_=*n*_1_/*m*. Thus, the probability that *v* of out-degree *k* in *V_T._* has no neighboring node of in-degree 1 is given by . Therefore, a lower bound of the probability that a node of out-degree *k* is a critical node is given by

. (Eq. 2)

On the other hand, we need two degree 1 neighbors in the case of unipartite undirected networks. In such a case, the corresponding probability is at least

, (Eq. 3)

where *p*_1_ in Eq. 3 is for unipartite networks. Eq. 2 gives a larger value than Eq. 3, which suggests that there exist more critical nodes in bipartite networks than in unipartite networks.

Table 3 shows the value of Eq. 2 and the actual fraction of critical nodes in the ncRNA-protein interaction network for each out-degree *k*. Eq. 2 gives good estimates for low out-degree nodes, whereas it does not for larger out-degree nodes, due to degree correlations or heterogeneity. If there exist degree correlations or some nodes connecting with many in-degree 1 nodes　(both Figs.1 and 2 suggest such possibilities), Eq. 2 does not necessarily give good estimates.

Many nodes of in-degree 1 are not critical. This tendency might be explained by the following proposition, which suggests that *p*_1_ is also the probability that a node of out-degree 1 is critical.

**Proposition 2.** Let *v* in *V_T_* be a node of out-degree 1. Then, *v* is a critical node if and only if *u* in *V_B_* has in-degree 1, where .

1. **DATA ANALYSIS**

**Network structure of the ncRNA-protein interaction network**

Using the NPInter v2.0 database, we extracted the molecular interactions corresponding to ncRNAs and proteins in humans, which led to a large bipartite network composed of 3,894 ncRNAs, 5,783 proteins and 92,998 interactions between ncRNAs and proteins. A visual representation of the entire network is shown in Fig. 1. The NPInter database includes a variety number of non-coding RNAs classes. A total of 32 classes were involved in the construction of the ncRNA-protein interaction network for human. The color legend in Fig. 1 denotes each main ncRNA class and Table 1 shows the statistics of each class. The miRNA class is the third largest class including 796 molecules, after the lncRNAs related classes, and it exhibits the highest average degree. One possible reason for these unbalanced degree values is that miRNAs have been studied in more detail than newly discovered ncRNA classes and their interactions and disease associations have been studied more systematically. Indeed, Fig. 1 also illustrates that the largest fraction of the interactions corresponds to miRNAs, namely the miRNA target interaction and regulatory class, which includes 85,355 (yellow) edges. A second large group is composed of 8,162 interactions (green) edges and is associated with the ncRNA-protein binding class. Other small groups of interaction classes, such as expression correlation with only 27 interactions, are denoted in grey in Fig. 1.

To analyze the global structure of the bipartite ncRNA-protein interaction network, we used the degree distribution. We considered unidirectional bipartite networks in which the direction is assigned from ncRNAs (set of top nodes) to proteins (set of bottom nodes). Hence, we can characterize the network by considering in-degree and out-degree distributions. The in-degree of a node (protein) *j* is the total number of connections onto node *j.* The out-degree of a node (ncRNA) *i*  is the total number of connections coming from node *i.* Then, the in-degree distribution, , is the fraction of nodes in the network with in-degree  and the out-degree distribution, , is the fraction of nodes with out-degree .

Because in some cases the degree distribution is noisy, in particular when the highest degree nodes appear as a few dots on the far right side of the tail, it is appropriate to compute the cumulative degree distribution, which indicates the fraction of vertices with degree greater than *k*. When the cumulative degree distribution follows a power-law , the associated degree distribution *P(k)* also follows a power-law with exponent . By using the collected datasets from NPInter v2.0 database, we plot the degree distributions for the ncRNA-protein network. The results shown in Figs. 2 (d-e) indicate that the protein degree distribution has a range of several decades compatible with a power-law distribution from and characterized by a degree exponent . The degree distribution for the ncRNAs shown in Figs. 2(a-c) suggests a more complicated picture. As shown in Table 1, the component related to miRNAs is highly connected and its degree distribution analysis reveals that it tends to decay exponentially. In contrast, the rest of the ncRNAs are less densely connected to proteins (see Table 1) and their degree distribution tends to follow a power-law decay for low degrees. The asymmetric degree distributions of these two large components of the same network are highlighted in Figs. 2(a-b). The explained tendency is more evident when the cumulative degree distribution is plotted on a log-linear scale, showing that, only from high degrees above *k>10,* the distribution follows an exponential decay of the form with . Three main findings can be derived from this analysis. First, there is a nonzero probability to find highly connected proteins interacting with ncRNAs (see Fig. 2 (d-e)). Second, a large fraction of ncRNAs interact with a similar number of proteins. Third, the unveiled structure of the ncRNA-protein interaction network displays an uncommon topology, characterized by two connected but drastically different sub-networks, one led by miRNAs and the other consisting of the rest of ncRNAs, mainly dominated by long ncRNAs (lncRNAs). By only removing 11 proteins (highlighted by a star symbol in Fig. 1), both large sub-networks become topologically disconnected. The biological functionality of the set of these ncRNA-bridges related proteins are shown in Table 2. Because the tendency of the protein degree distribution is a power-law, there should be a small set of highly connected proteins. The degree of each hub is also shown in Table 2. Most of the highly connected proteins are related to lncRNA, and low degree proteins tend to be associated to the miRNA component.

**Enrichment of critical and redundant nodes in ncRNA disease associations**

Annotations regarding diseases associations from the HMDD database resources were mapped to the ncRNAs obtained from the NPInter database. We then classified the ncRNAs into two groups based on whether they have disease or non-disease association. Another classification was performed for ncRNAs based on whether they belong to the MDS and the critical set of nodes. Among all possible MDS configurations, we selected one and classified the ncRNAs as shown in Table S1 as a contingency table. The total number of different diseases included in the database is 367. By using the results shown in Table S1, we applied Fisher’s exact test to determine whether the MDS of ncRNAs is significantly enriched with disease associations. The result of the test was a two-tailed exact P-value with a strong signal . Therefore, the associations between disease and ncRNAs that belong to the MDS are statistically significant.

Because the MDS is not unique, we focus on those critical nodes that are always engaged in network control. The results for the critical set of ncRNAs are shown in Table S1. Applying Fisher’s exact test we found that diseases were significantly enriched in the critical set of ncRNAs with a two-tailed exact P-value of . A histogram with the number of ncRNAs that play a critical role in network control and associated to each disease is shown in Fig. S2. Out of all diseases, the histogram only shows data for the top 28 diseases with the highest number of ncRNA engaged in critical network control and associated with the disease. The histogram is dominated by hepatocellular carcinoma, and stomach, breast and colorectal neoplasms.

Next, we investigated the enrichment of the MDS and the critical set of ncRNAs for each particular disease. The results for the top 30 diseases with highest number of ncRNA associations are shown in Fig. 5. Next to the enrichment scores, the two-tailed P-values for the Fisher’s exact test are displayed. A full list with all diseases is shown in Table S2 (Excel file). The result demonstrates that for each disease there is a significant enrichment in both MDS and the critical set. When only diseases that passed the Fisher’s exact test are considered, the enrichment of critical set is, in most cases, higher than that of the MDS (Fig. S3), which reinforces the importance in network control. Moreover, the enrichment function does not depend strongly on the size of the MDS or the critical set involved in the disease and, on average, is distributed at approximately 0.5 (Fig. S4).

We also investigated the number of ncRNAs, excluding miRNAs, involved in diseases using different repository resources such as the lncRNADisease database [12]. The resulting number of the ncRNAs that are present in our network and that are also associated to diseases in the lncRNA database is not high. Only 31 ncRNAs, mainly extracted from 7SK RNA, H19 RNA, IPW RNA, MESTIT1 RNA, SRP RNA, XIST RNA, mRNAlike lncRNA and lncRNA classes, were mapped to our network. Here the lncRNA-related classes showed the highest representation. Combining this data with the HMDD database, we computed again the statistical significance of ncRNA associations with diseases (see Table S3). Applying Fisher’s exact test we found that diseases were significantly enriched in the MDS of the ncRNAs with a two-tailed exact P-value of and also in the critical set of the ncRNAs with two-tailed exact P-value of .

Finally, we investigated the ncRNA-disease associations reported in the OMiR dataset. The total number of diseases included in the database is 79. By applying the Fisher’s exact test to the data shown in Table S4, we found that the MDS of the ncRNAs is enriched with orphan diseases . We hypothesized that the critical set of the ncRNAs is also enriched with disease associations. The Fisher’s exact test result showed a statistically significant association between the critical set of the ncRNAs and the set of “orphan” Mendelian diseases . In contrast to the HMDD database, the OMiR dataset consists of single-gene diseases. These diseases were represented in a histogram based on the number of ncRNAs that play a critical role in the network control for each disease as shown in Fig. S5. Cataract, coronary heart disease, gene expression variation in chromosome 14, hemifacial microsomia and microphthalmia are the top five diseases with a higher number of associated critical ncRNAs. These five disorders are associated with 50% more critical nodes than the next lower ranked disease.

**SI References**

[1] Li, Y. et al. HMDD v2.0: a database for experimentally supported human microRNA and disease associations. *Nucl. Acids Res.* 42: D1070-4 (2014).

[2] Rossi, S., et al. OMiR: Identification of associations between OMIM diseases and microRNAs. *Genomics* **97**, 71-76 (2011).

[3] OMIM, Online Mendelian Inheritance in Man, OMIM®. McKusick-Nathans Institute of Genetic Medicine, Johns Hopkins University (Baltimore, MD) (2014)

{Accessed: 2014-06-15.}. World Wide Web URL: <http://omim.org/>

[4] Nacher, J. C. & Akutsu, T. Structural controllability of unidirectional bipartite networks, *Scientific Reports* **3**, 1647 (2013).

[5] Johnson, D. S. Approximation algorithms for combinatorial problems. *Journal of Computer and System Sciences* **9**, 256–278 (1974).

[6] Chvátal, V. A greedy heuristic for the set-covering problem. *Mathematics of Operations Research* **4**, 233–235 (1979).

[7] Lovász, L. On the ratio of optimal integral and fractional covers. *Discrete Mathematics* **13**, 383–390 (1975).

[8] Haynes, T. W. Hedetniemi, S. T. & Slater, P. J. Fundamentals of Domination in graphs (Chapman and Hall/CRC Pure Applied Mathematics, New York, 1998).

[9] Amaldi, E., Capone, A., Malucelli, F. & Mannino, C. Optimization problems and models for planning cellular networks (Handbook of Optimization in Telecommunications, 2006).

[10] Nacher, J. C. & Akutsu, T. Analysis of critical and redundant nodes in controlling directed and undirected complex networks using dominating sets. *Journal of Complex Networks,* **2***,* 394-412 (2014).

[11] Wuchty, S. Controllability in protein interaction networks. *Proc. Natl. Acad. Sci.* **111,** 7156-7160 (2014).

[12] Chen, G. et al. LncRNADisease: a database for long-non-coding RNA-associated diseases. *Nucleic Acids Res.* **41**, D983-6 (2013).

**Supplementary figures and tables**

**
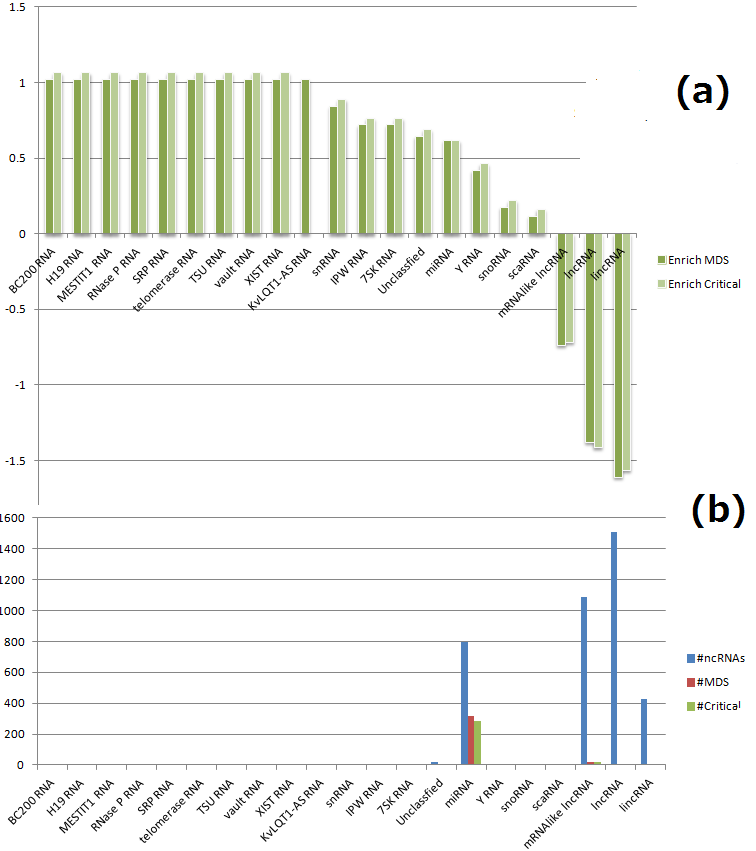
**

**Figure S1 - Enrichment of MDS and critical set per ncRNA classes and size of ncRNA classes** (a) Enrichment of MDS and critical set in each nRNA class.

Although most classes are populated by few ncRNAs, some classes contain many molecules. Among the latter, miRNAs show the highest enrichment. The largest classes, which are related to lncRNAs, show a depletion in controllability roles. (b) Histogram with the number of ncRNAs in each class.


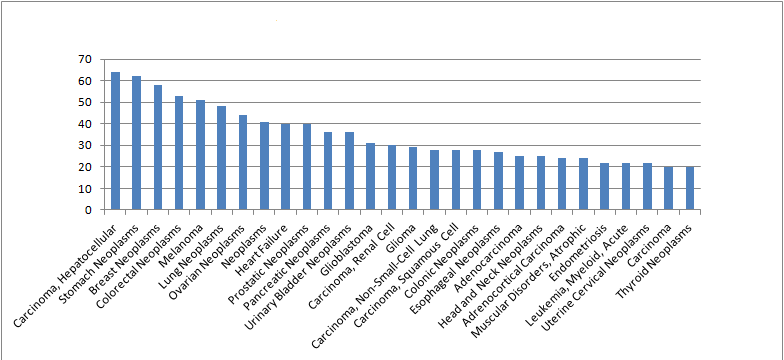


**Figure S2 - Histogram for critical ncRNAs associated with polygenic diseases.**

Each histogram bar corresponds to a disease and indicates the number of ncRNAs that play critical role in network control and are also associated with the disease from the HMDD database. Out of all diseases, the histogram only shows data for the top 28 diseases with the highest number of ncRNAs engaged in critical network control.


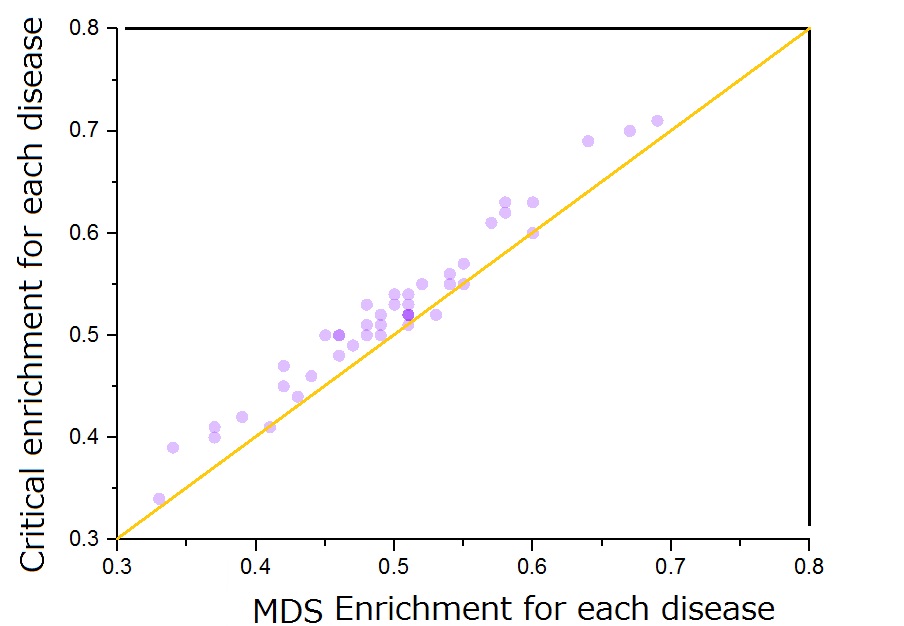


**Figure S3 - Relation between the MDS and critical enrichment for each disease**

Each dot represents a disease with an MDS and critical enrichment score. Critical enrichment tends to be higher than MDS enrichment. Only diseases with the highest statistical significance (two-tailed p-value below 0.05) are considered.


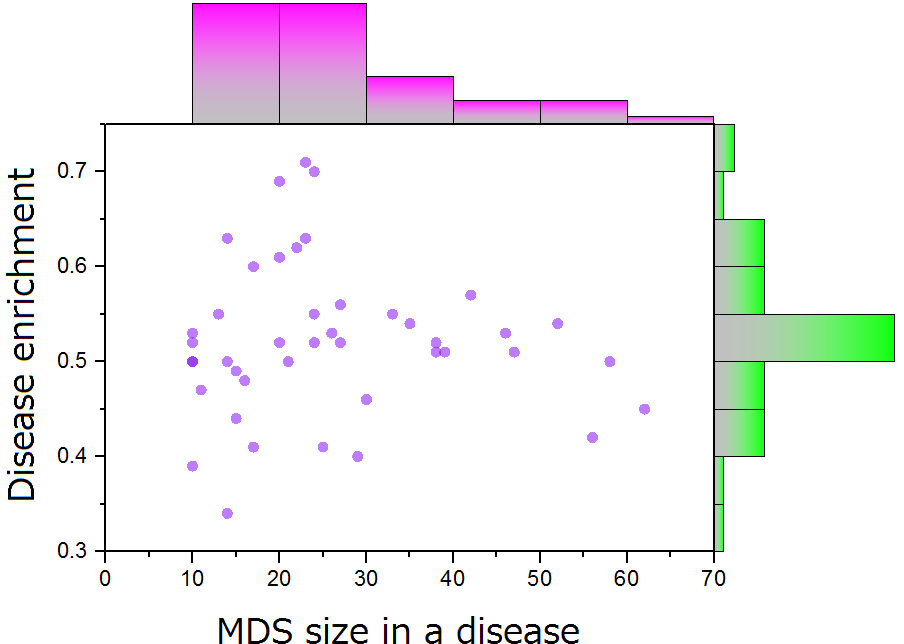


**Figure S4 - Dependence of the MDS enrichment with the MDS size for each disease.**

Each dot represents a disease. The enrichment does not significantly change with the increasing statistics of the MDS size for each disease and is homogeneously distributed at approximately 0.5. Only diseases with the highest statistical significance (two-tailed p-value below 0.05) are considered.


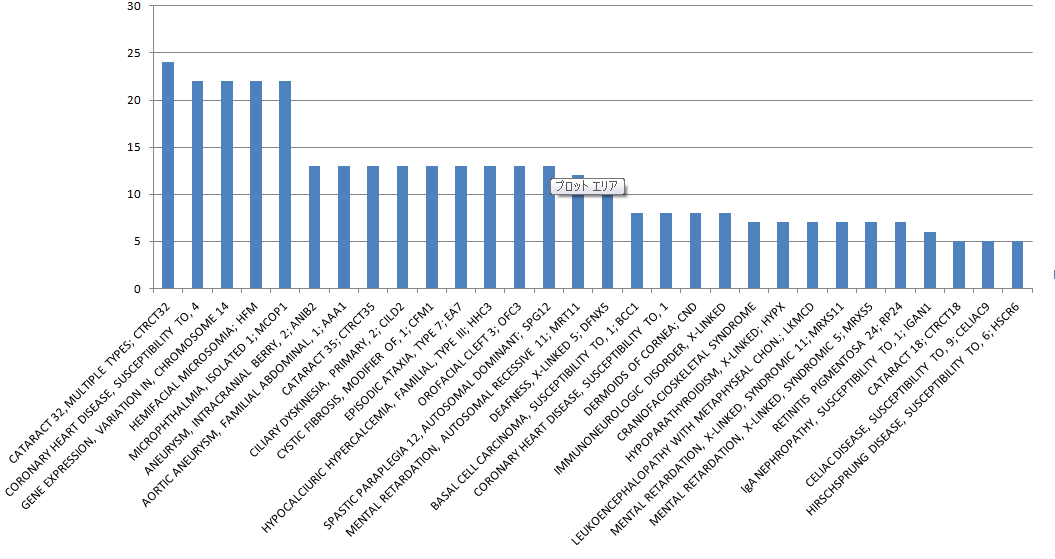


**Figure S5 - Histogram for critical ncRNAs associated with ‘rare’ or single-gene diseases.**

Each histogram bar corresponds to a disease and indicates the number of ncRNAs that play a critical role in network control and are also associated with the disease in the OMiR database. The histogram only shows data for the top 30 diseases with the highest number of ncRNAs engaged in critical network control.

| **HMDD** |  |  |  |
| --- | --- | --- | --- |
|  | MDS | non-MDS | Total |
| **Disease** | **140** | **28** | 168 |
| **non-Disease** | **231** | **3495** | 3726 |
|  | 371 | 3523 | 3894 |
|  |  |  |  |
|  |  |  |  |
|  | Critical | non-Critical | Total |
| **Disease** | **127** | **41** | 168 |
| **non-Disease** | **208** | **3518** | 3726 |
|  | 335 | 3559 | 3894 |

**Table S1 - Results of the MDS and critical set for the ncRNAs from the HMDD database**

This table shows the number of ncRNAs that belong (or not) to an MDS (upper) and critical set (lower) and are also associated (or not) with more than one disease. Associations between ncRNAs and diseases are obtained from the HMDD database.

|  |  |  | **TABLE S2** |  |  |
| --- | --- | --- | --- | --- | --- |
| **Number of ncrnas** | **Disease** | **MDS Enrichment** | Fischer Exact Test (Two-tailed P-value) | **Critical Enrichment** | Fischer Exact Test  (Two-tailed P-value) |
| **250** | Carcinoma, Hepatocellular | 0.42 | 2.26E-11 | 0.45 | 2.22E-10 |
| **243** | Breast Neoplasms | 0.39 | 1.85E-10 | 0.42 | 4.43E-10 |
| **210** | Stomach Neoplasms | 0.49 | 1.17E-09 | 0.5 | 8.60E-09 |
| **171** | Colorectal Neoplasms | 0.51 | 5.84E-08 | 0.54 | 2.93E-07 |
| **165** | Melanoma | 0.51 | 1.06E-07 | 0.51 | 5.02E-07 |
| **157** | Lung Neoplasms | 0.5 | 1.06E-07 | 0.53 | 1.03E-06 |
| **137** | Heart Failure | 0.48 | 1.70E-06 | 0.51 | 6.15E-06 |
| **134** | Prostatic Neoplasms | 0.49 | 2.28E-06 | 0.51 | 8.04E-06 |
| **133** | Carcinoma, Renal Cell | 0.37 | 1.84E-05 | 0.4 | 1.48E-05 |
| **131** | Neoplasms | 0.51 | 3.06E-06 | 0.52 | 1.05E-05 |
| **129** | Ovarian Neoplasms | 0.55 | 3.73E-06 | 0.57 | 1.25E-05 |
| **120** | Glioblastoma | 0.44 | 9.30E-06 | 0.46 | 2.82E-05 |
| **117** | Pancreatic Neoplasms | 0.5 | 1.22E-05 | 0.54 | 3.64E-05 |
| **113** | Carcinoma, Non-Small-Cell Lung | 0.41 | 2.37E-05 | 0.41 | 7.32E-05 |
| **106** | Urinary Bladder Neoplasms | 0.55 | 3.57E-05 | 0.55 | 9.66E-05 |
| **93** | Carcinoma, Squamous Cell | 0.49 | 1.28E-04 | 0.52 | 3.04E-04 |
| **89** | Colonic Neoplasms | 0.51 | 1.88E-04 | 0.53 | 3.04E-04 |
| **86** | Glioma | 0.54 | 1.88E-04 | 0.56 | 4.33E-04 |
| **83** | Esophageal Neoplasms | 0.53 | 3.38E-04 | 0.52 | 7.34E-04 |
| **78** | Head and Neck Neoplasms | 0.52 | 5.49E-04 | 0.55 | 1.14E-03 |
| **76** | Leukemia, Myeloid, Acute | 0.48 | 6.73E-04 | 0.5 | 1.36E-03 |
| **76** | Medulloblastoma | 0.37 | 1.87E-03 | 0.41 | 1.75E-03 |
| **73** | Adenoviridae Infections | 0.33 | 5.86E-03 | 0.34 | 6.67E-03 |
| **70** | Endometriosis | 0.51 | 1.23E-03 | 0.52 | 2.30E-03 |
| **62** | Adrenocortical Carcinoma | 0.6 | 2.59E-03 | 0.63 | 4.62E-03 |
| **62** | Endometrial Neoplasms | 0.43 | 2.97E-03 | 0.44 | 5.01E-03 |
| **61** | Lupus Vulgaris | 0.46 | 2.96E-03 | 0.48 | 5.15E-03 |
| **60** | Uterine Cervical Neoplasms | 0.58 | 3.15E-03 | 0.62 | 5.51E-03 |
| **56** | Lymphoma | 0.47 | 4.76E-03 | 0.49 | 7.94E-03 |
| **56** | Thyroid Neoplasms | 0.57 | 4.64E-03 | 0.61 | 7.81E-03 |
| **55** | Adenocarcinoma | 0.67 | 5.11E-03 | 0.7 | 8.52E-03 |
| **51** | Muscular Disorders, Atrophic | 0.69 | 7.51E-03 | 0.71 | 1.21E-02 |
| **51** | Osteosarcoma | 0.45 | 7.99E-03 | 0.5 | 1.23E-02 |
| **49** | Leukemia, Lymphocytic, Chronic, B-Cell | 0.6 | 9.11E-03 | 0.6 | 1.44E-02 |
| **47** | Carcinoma | 0.64 | 1.11E-02 | 0.69 | 1.71E-02 |
| **47** | Neuroblastoma | 0.34 | 2.70E-02 | 0.39 | 2.54E-02 |
| **44** | Nasopharyngeal Neoplasms | 0.33 |  | 0.32 |  |
| **44** | Retinoblastoma | 0.22 |  | 0.2 |  |
| **43** | Myocardial Infarction | 0.42 | 1.94E-02 | 0.47 | 2.40E-02 |
| **42** | Myelodysplastic Syndromes | 0.54 | 1.80E-02 | 0.55 | 2.65E-02 |
| **41** | Schizophrenia | 0.4 | 2.77E-02 | 0.35 |  |
| **40** | Leukemia | 0.41 | 2.73E-02 | 0.41 |  |
| **38** | Autistic Disorder | 0.58 | 2.63E-02 | 0.63 | 3.74E-02 |
| **37** | Hepatitis C | 0.53 | 2.92E-02 | 0.45 |  |
| **36** | Neoplasms, Squamous Cell | 0.46 | 3.46E-02 | 0.5 | 4.58E-02 |
| **36** | Pre-Eclampsia | 0.46 | 3.46E-02 | 0.5 | 4.58E-02 |
| **35** | Mesothelioma | 0.51 | 3.69E-02 | 0.52 | 4.96E-02 |
| **35** | Parkinson Disease | 0.32 |  | 0.29 |  |
| **34** | Hepatitis B | 0.48 | 4.05E-02 | 0.53 | 5.38E-02 |
| **34** | Lymphoma, B-Cell | 0.6 | 3.87E-02 | 0.57 | 5.32E-02 |
| **34** | Multiple Myeloma | 0.63 | 3.87E-02 | 0.64 | 5.30E-02 |
| **34** | Periodontitis | 0.09 |  | 0.13 |  |
| **32** | Carcinoma, Basal Cell | 0.29 |  | 0.25 |  |
| **32** | Gastrointestinal Neoplasms | 0.66 | 4.68E-02 | 0.7 | 5.30E-02 |
| **32** | Tuberculosis, Pulmonary | 0.36 |  | 0.4 |  |
| **31** | Inflammation | 0.64 | 5.16E-02 | 0.68 | 6.87E-02 |
| **30** | Alzheimer Disease | 0.32 |  | 0.36 |  |
| **29** | Eosinophilic Esophagitis | 0.46 |  | 0.5 |  |
| **29** | Laryngeal Neoplasms | 0.6 | 6.26E-02 | 0.64 | 8.18E-02 |
| **29** | Lupus Erythematosus, Systemic | 0.73 | 6.25E-02 | 0.74 | 8.18E-02 |
| **28** | Hodgkin Disease | 0.27 |  | 0.31 |  |
| **28** | Mouth Neoplasms | 0.57 | 6.92E-02 | 0.57 |  |
| **27** | Hypertrophy | 0.54 |  | 0.58 |  |
| **26** | Multiple Sclerosis | 0.45 |  | 0.42 |  |
| **25** | Atherosclerosis | 0.66 | 9.17E-02 | 0.7 | 1.16E-01 |
| **25** | Rectal Neoplasms | 0.1 |  | 0.14 |  |
| **24** | Myotonic Dystrophy | 0.54 |  | 0.53 |  |
| **24** | Precursor Cell Lymphoblastic Leukemia-Lymphoma | 0.54 |  | 0.46 |  |
| **24** | Salivary Gland Neoplasms | 0.68 | 1.03E-01 | 0.63 |  |
| **23** | Cardiomyopathy, Hypertrophic | 0.43 |  | 0.48 |  |
| **22** | Astrocytoma | 0.52 |  | 0.56 |  |
| **22** | Hypertension | 0.52 |  | 0.56 |  |
| **22** | Liver Cirrhosis | 0.58 |  | 0.56 |  |
| **21** | Asthma | 0.6 |  | 0.64 |  |
| **20** | Arthritis, Rheumatoid | 0.49 |  | 0.54 |  |
| **20** | Atrial Fibrillation | 0.41 |  | 0.46 |  |
| **20** | Azoospermia | 0.41 |  | 0.46 |  |
| **20** | Barrett Esophagus | 0.79 |  | 0.84 |  |
| **20** | Coronary Artery Disease | 0.56 |  | 0.6 |  |
| **20** | Crohn Disease | 0.62 |  | 0.6 |  |
| **19** | Diabetes Mellitus, Type 2 | 0.69 |  | 0.74 |  |
| **19** | Liver Neoplasms | 0.44 |  | 0.48 |  |
| **19** | Sarcoma, Kaposi | 0.52 |  | 0.56 |  |
| **18** | Leukemia-Lymphoma, Adult T-Cell | 0.36 |  | 0.41 |  |
| **18** | Pituitary Neoplasms | 0.54 |  | 0.58 |  |
| **17** | Lymphoma, T-Cell | 0.09 |  | 0.13 |  |
| **17** | Psoriasis | 0.56 |  | 0.61 |  |
| **17** | Pulmonary Disease, Chronic Obstructive | 0.09 |  | 0.13 |  |
| **17** | Stroke | 0.26 |  | 0.31 |  |
| **16** | Leukemia, Myeloid | 0.51 |  | 0.56 |  |
| **16** | Polycystic Kidney Diseases | 0.29 |  | 0.33 |  |
| **16** | Prolactinoma | 0.59 |  | 0.63 |  |
| **16** | Sepsis | 0.29 |  | 0.33 |  |
| **15** | Aortic Aneurysm, Thoracic | 0.54 |  | 0.36 |  |
| **15** | Biliary Tract Neoplasms | 0.54 |  | 0.58 |  |
| **15** | Cholangiocarcinoma | 0.79 |  | 0.84 |  |
| **15** | Sarcoma, Ewing | 0.44 |  | 0.49 |  |
| **15** | Scleroderma, Systemic | 0.54 |  | 0.58 |  |
| **14** | Ischemia | 0.71 |  | 0.76 |  |
| **14** | Leukemia, Myelogenous, Chronic, BCR-ABL Positive | 0.57 |  | 0.61 |  |
| **14** | Lymphoma, Primary Effusion | 0.57 |  | 0.61 |  |
| **14** | Muscular Dystrophy, Duchenne | 0.47 |  | 0.39 |  |
| **14** | Reperfusion Injury | 0.77 |  | 0.82 |  |
| **13** | Carcinoma, Small Cell | 0.38 |  | 0.42 |  |
| **13** | Gout | 0.2 |  | 0.25 |  |
| **13** | Hepatitis, Chronic | 0.75 |  | 0.79 |  |
| **13** | Myocytes, Cardiac | 0.38 |  | 0.42 |  |
| **12** | Cystitis, Interstitial | 0.24 |  | 0.28 |  |
| **12** | Leukemia, B-Cell | 0.54 |  | 0.58 |  |
| **11** | ACTH-Secreting Pituitary Adenoma | 0.58 |  | 0.62 |  |
| **11** | Diabetic Nephropathies | 0.82 |  | 0.8 |  |
| **11** | Digestive System Neoplasms | 0.45 |  | 0.5 |  |
| **11** | HIV | 0.58 |  | 0.62 |  |
| **11** | Lung Diseases, Interstitial | 0.67 |  | 0.62 |  |
| **11** | Lymphoma, Large-Cell, Anaplastic | 0.45 |  | 0.5 |  |
| **11** | Lymphoma, Mantle-Cell | 0.58 |  | 0.62 |  |
| **11** | Musculoskeletal Abnormalities | 0.45 |  | 0.5 |  |
| **11** | Polycythemia Vera | 0.58 |  | 0.62 |  |
| **10** | Brain Neoplasms | 0.32 |  | 0.36 |  |
| **10** | Burkitt Lymphoma | 0.71 |  | 0.76 |  |
| **10** | Muscular Dystrophies | 0.71 |  | 0.76 |  |
| **10** | Mycosis Fungoides | 0.49 |  | 0.54 |  |
| **10** | Rhabdomyosarcoma | 0.32 |  | 0.06 |  |
| **10** | Vascular Diseases | 0.86 |  | 0.91 |  |
| **9** | Cardiomegaly | 0.06 |  | 0.11 |  |
| **9** | Diabetes Mellitus | 0.96 |  | 0.95 |  |
| **9** | Ependymoma | 0.54 |  | 0.58 |  |
| **9** | Hematologic Neoplasms | 0.36 |  | 0.41 |  |
| **9** | Huntington Disease | 0.76 |  | 0.81 |  |
| **9** | Leiomyoma | 0.54 |  | 0.58 |  |
| **9** | Obesity | 0.76 |  | 0.71 |  |
| **9** | Retinal Degeneration | 0.06 |  | 0.11 |  |
| **9** | Rhinitis, Allergic, Perennial | 0.36 |  | 0.41 |  |
| **9** | Waldenstrom Macroglobulinemia | 0.66 |  | 0.71 |  |
| **8** | Acute Coronary Syndrome | 0.41 |  | 0.46 |  |
| **8** | Adrenal Cortex Neoplasms | 0.81 |  | 0.86 |  |
| **8** | Adrenocortical Adenoma | 0.81 |  | 0.86 |  |
| **8** | Cerebral Infarction | 0.11 |  | 0.16 |  |
| **8** | Colitis, Ulcerative | 0.81 |  | 0.86 |  |
| **8** | Helplessness, Learned | 0.81 |  | 0.86 |  |
| **8** | Lichen Planus, Oral | 0.71 |  | 0.76 |  |
| **8** | Lymphoma, Large B-Cell, Diffuse | 0.71 |  | 0.76 |  |
| **8** | Myocardium | 0.59 |  | 0.63 |  |
| **8** | Osteoarthritis | 0.41 |  | 0.46 |  |
| **8** | Spinal Cord Injuries | 0.41 |  | 0.46 |  |
| **8** | Testicular Neoplasms | 0.11 |  | 0.16 |  |
| **8** | Toxoplasmosis | 0.71 |  | 0.76 |  |
| **7** | Anxiety Disorders | 0.17 |  | 0.22 |  |
| **7** | Carcinoma, Ehrlich Tumor | 0.77 |  | 0.82 |  |
| **7** | Cocaine-Related Disorders | 0.17 |  | 0.22 |  |
| **7** | Fatty Liver | 0.65 |  | 0.69 |  |
| **7** | Hand, Foot and Mouth Disease | 0.47 |  | 0.52 |  |
| **7** | HBV Infection | 0.47 |  | 0.52 |  |
| **7** | Hepatoblastoma | 0.65 |  | 0.69 |  |
| **7** | HIV Infections | 0.17 |  | 0.22 |  |
| **7** | Inflammatory Bowel Diseases | 0.65 |  | 0.69 |  |
| **7** | Kidney Diseases | 0.65 |  | 0.52 |  |
| **7** | Kidney Neoplasms | 0.95 |  | 0.99 |  |
| **7** | Liver Failure | 0.47 |  | 0.52 |  |
| **7** | Lupus Nephritis | 0.47 |  | 0.52 |  |
| **7** | Meningioma | 0.77 |  | 0.69 |  |
| **7** | Myocardial Ischemia | 0.87 |  | 0.91 |  |
| **7** | Neurodegenerative Diseases | 0.47 |  | 0.52 |  |
| **7** | Pain | 0.47 |  | 0.52 |  |
| **7** | Precursor B-Cell Lymphoblastic Leukemia-Lymphoma | 0.65 |  | 0.69 |  |
| **7** | Retinal Neovascularization | 0.47 |  | 0.52 |  |
| **7** | Toxoplasma | 0.47 |  | 0.52 |  |
| **7** | Trophoblasts | 0.65 |  | 0.69 |  |
| **7** | Wounds and Injuries | 0.65 |  | 0.69 |  |
| **6** | Aging | 0.24 |  | 0.28 |  |
| **6** | Aortic Valve Stenosis | 0.71 |  | 0.76 |  |
| **6** | Cardiomyopathies | 0.71 |  | 0.76 |  |
| **6** | Cardiomyopathy, Dilated | 0.84 |  | 0.88 |  |
| **6** | Fibrosis | 0.71 |  | 0.58 |  |
| **6** | Glomerulonephritis, IGA | 0.94 |  | 0.98 |  |
| **6** | Heart Defects, Congenital | 0.54 |  | 0.58 |  |
| **6** | Hemangiosarcoma | 0.54 |  | 0.58 |  |
| **6** | HPV Infection | 0.71 |  | 0.58 |  |
| **6** | Kidney Failure, Chronic | 0.84 |  | 0.88 |  |
| **6** | Muscular Dystrophy, Facioscapulohumeral | 0.84 |  | 0.88 |  |
| **6** | Myeloproliferative Disorders | 0.71 |  | 0.76 |  |
| **6** | Nephrosclerosis | 0.84 |  | 0.88 |  |
| **6** | Prostate Neoplasms | 0.24 |  | 0.28 |  |
| **5** | Alopecia | 0.32 |  | 0.36 |  |
| **5** | Cerebral Hemorrhage | 0.62 |  | 0.66 |  |
| **5** | Cholesteatoma | 0.62 |  | 0.66 |  |
| **5** | Distal Myopathies | 0.32 |  | 0.36 |  |
| **5** | Eclampsia | 0.92 |  | 0.96 |  |
| **5** | Endomyocardial Fibrosis | 0.62 |  | 0.66 |  |
| **5** | Hypertrophy, Left Ventricular | 0.62 |  | 0.66 |  |
| **5** | Leukemia, Myeloid, Chronic-Phase | 0.32 |  | 0.36 |  |
| **5** | Liver Diseases | 0.32 |  | 0.36 |  |
| **5** | Periodontal Diseases | 0.79 |  | 0.66 |  |
| **5** | PRRSV Infection | 0.32 |  | 0.36 |  |
| **4** | Aortic Valve Insufficiency | 0.71 |  | 0.76 |  |
| **4** | Child Development Disorders, Pervasive | 0.41 |  | 0.46 |  |
| **4** | Colon Neoplasms | 0.71 |  | 0.76 |  |
| **4** | Cryptosporidium | 0.89 |  | 0.94 |  |
| **4** | Fatty Liver, Non-Alcoholic | 0.41 |  | 0.46 |  |
| **4** | Gastric Neoplasms | 0.89 |  | 0.94 |  |
| **4** | Heart Diseases | 0.89 |  | 0.94 |  |
| **4** | HIV-1 | 0.41 |  | 0.46 |  |
| **4** | Influenza, Human | 0.71 |  | 0.46 |  |
| **4** | Liposarcoma | 0.71 |  | 0.76 |  |
| **4** | Marek Disease | 0.71 |  | 0.76 |  |
| **4** | Panic Disorder | 0.71 |  | 0.76 |  |
| **4** | Sezary Syndrome | 0.71 |  | 0.76 |  |
| **4** | Skin Neoplasms | 0.41 |  | 0.46 |  |
| **4** | Tongue Neoplasms | 0.89 |  | 0.94 |  |
| **3** | Acquired Immunodeficiency Syndrome | 0.54 |  | 0.58 |  |
| **3** | Atrophy | 0.54 |  | 0.58 |  |
| **3** | Carcinoma, Endometrioid | 1.02 |  | 1.06 |  |
| **3** | Dementia | 0.54 |  | 0.58 |  |
| **3** | Demyelinating Diseases | 1.02 |  | 1.06 |  |
| **3** | Dermatitis, Atopic | 0.54 |  | 0.58 |  |
| **3** | Graves Disease | 1.02 |  | 1.06 |  |
| **3** | Leukemia, Biphenotypic, Acute | 0.84 |  | 0.58 |  |
| **3** | Lymphoma, Non-Hodgkin | 0.84 |  | 0.88 |  |
| **3** | Odontogenic Tumors | 0.54 |  | 0.58 |  |
| **3** | Patau Syndrome | 0.54 |  | 0.58 |  |
| **3** | Sarcoma, Synovial | 0.84 |  | 0.88 |  |
| **3** | SARS Virus | 0.54 |  | 0.58 |  |
| **3** | Small Cell Lung Carcinoma | 0.54 |  | 0.58 |  |
| **3** | Vascular Calcification | 0.54 |  | 0.58 |  |
| **2** | AIDS Dementia Complex | 0.71 |  | 0.76 |  |
| **2** | Antiphospholipid Syndrome | 0.71 |  | 0.76 |  |
| **2** | Arthritis | 0.71 |  | 0.76 |  |
| **2** | Arthritis, Psoriatic | 1.02 |  | 1.06 |  |
| **2** | Behcet Syndrome | 1.02 |  | 1.06 |  |
| **2** | Brain Injuries | 1.02 |  | 1.06 |  |
| **2** | Burns | 0.71 |  | 0.76 |  |
| **2** | Carcinoma, Neuroendocrine | 1.02 |  | 1.06 |  |
| **2** | Cardiovascular Diseases | 0.71 |  | 0.76 |  |
| **2** | Carotid Artery Diseases | 1.02 |  | 1.06 |  |
| **2** | Cerebellar Neoplasms | 0.71 |  | 0.76 |  |
| **2** | Choriocarcinoma | 0.71 |  | 0.76 |  |
| **2** | Down Syndrome | 0.71 |  | 0.76 |  |
| **2** | Drug-Induced Liver Injury | 0.71 |  | 0.76 |  |
| **2** | Esophagus | 0.71 |  | 0.76 |  |
| **2** | Fibrosarcoma | 0.71 |  | 0.76 |  |
| **2** | Hamartoma Syndrome, Multiple | 1.02 |  | 1.06 |  |
| **2** | HEV | 1.02 |  | 1.06 |  |
| **2** | Hyperlipidemias | 0.71 |  | 0.76 |  |
| **2** | Lymphoma, Extranodal NK-T-Cell | 1.02 |  | 1.06 |  |
| **2** | Moyamoya Disease | 0.71 |  | 0.76 |  |
| **2** | Myocardial Reperfusion Injury | 1.02 |  | 1.06 |  |
| **2** | Neoplasms, Germ Cell and Embryonal | 0.71 |  | 0.76 |  |
| **2** | Neutropenia | 1.02 |  | 0.76 |  |
| **2** | Papilary thyroid carcinoma | 1.02 |  | 1.06 |  |
| **2** | Prion Diseases | 1.02 |  | 1.06 |  |
| **2** | Psychotic Disorders | 0.71 |  | 0.76 |  |
| **2** | Pulmonary Fibrosis | 1.02 |  | 1.06 |  |
| **2** | Sjogren's Syndrome | 0.71 |  | 0.76 |  |
| **2** | Stomach Diseases | 0.71 |  | 0.76 |  |
| **1** | Adenoma | 1.02 |  | 1.06 |  |
| **1** | Albuminuria | 1.02 |  | 1.06 |  |
| **1** | Amyotrophic Lateral Sclerosis | 1.02 |  | 1.06 |  |
| **1** | Angina, Unstable | 1.02 |  | 1.06 |  |
| **1** | Anus Neoplasms | 1.02 |  | 1.06 |  |
| **1** | Aortic Aneurysm, Abdominal | 1.02 |  | 1.06 |  |
| **1** | Carcinoma, Ductal, Breast | 1.02 |  | 1.06 |  |
| **1** | Carcinoma, Oral | 1.02 |  | 1.06 |  |
| **1** | Chlamydia Infections | 1.02 |  | 1.06 |  |
| **1** | Colitis | 1.02 |  | 1.06 |  |
| **1** | Colorectal Neoplasms, Hereditary Nonpolyposis | 1.02 |  | 1.06 |  |
| **1** | Creutzfeldt-Jakob Syndrome | 1.02 |  | 1.06 |  |
| **1** | Cystic Fibrosis | 1.02 |  | 1.06 |  |
| **1** | Diabetes Complications | 1.02 |  | 1.06 |  |
| **1** | Diabetic Retinopathy | 1.02 |  | 1.06 |  |
| **1** | Eczema | 1.02 |  | 1.06 |  |
| **1** | Encephalomyelitis, Autoimmune, Experimental | 1.02 |  | 1.06 |  |
| **1** | Endothelium, Vascular | 1.02 |  | 1.06 |  |
| **1** | Fanconi Anemia | 1.02 |  | 1.06 |  |
| **1** | Fibroblasts | 1.02 |  | 1.06 |  |
| **1** | Focal Epithelial Hyperplasia | 1.02 |  | 1.06 |  |
| **1** | Francisella | 1.02 |  | 1.06 |  |
| **1** | Gastritis, Atrophic | 1.02 |  | 1.06 |  |
| **1** | Gerstmann-Straussler-Scheinker Disease | 1.02 |  | 1.06 |  |
| **1** | Giant Cell Tumors | 1.02 |  | 1.06 |  |
| **1** | Graft vs Host Disease | 1.02 |  | 1.06 |  |
| **1** | Granulosa Cell Tumor | 1.02 |  | 1.06 |  |
| **1** | Hyperglycemia | 1.02 |  | 1.06 |  |
| **1** | Hypopharyngeal Neoplasms | 1.02 |  | 1.06 |  |
| **1** | Hypoxia-Ischemia, Brain | 1.02 |  | 1.06 |  |
| **1** | Intervertebral Disk | 1.02 |  | 1.06 |  |
| **1** | Leiomyosarcoma | 1.02 |  | 1.06 |  |
| **1** | Leprosy | 1.02 |  | 1.06 |  |
| **1** | Leukemia, Acute | 1.02 |  | 1.06 |  |
| **1** | Leukemia, lymphoblastic, Acute | 1.02 |  | 1.06 |  |
| **1** | Leukoplakia, Oral | 1.02 |  | 1.06 |  |
| **1** | Myopia | 1.02 |  | 1.06 |  |
| **1** | Neuroma, Acoustic | 1.02 |  | 1.06 |  |
| **1** | Osteolysis | 1.02 |  | 1.06 |  |
| **1** | Ovary Syndrome | 1.02 |  | 1.06 |  |
| **1** | Renal Insufficiency | 1.02 |  | 1.06 |  |
| **1** | RNA Virus Infections | 1.02 |  | 1.06 |  |
| **1** | Schistosomiasis | 1.02 |  | 1.06 |  |
| **1** | Synapses | 1.02 |  | 1.06 |  |

**Table S2 Enrichment of the MDS and critical set for each disease**

The results of all diseases were classified by the number of ncRNA associations. Next to the enrichment scores for the MDS and critical set, the two-tailed P-values for the Fisher’s exact test are displayed. For statistical significance tests, a two tailed p-value of more (less) than 0.05 rejects (accepts) the hypothesis of disease association with the MDS and critical set. For rejected cases, the p-value is shown in blue. We are applying a two-tailed p-value test, which is always stricter than a one-tailed p-value test. For statistical reasons, we only computed the statistical tests when the sample was enough large. That is, when the number of ncRNAs associated with a given disease *D* that appeared in the MDS or Critical set was equal to or greater than 10 and the number of total ncRNAs that are in the MDS or Critical set was equal or greater than (*10 +* ).

| **HMDD+LNCRNA** |  |  |  |
| --- | --- | --- | --- |
|  | MDS | non-MDS | Total |
| **Disease** | **165** | **34** | 199 |
| **non-Disease** | **206** | **3489** | 3695 |
|  | 371 | 3523 | 3894 |
|  |  |  |  |
|  |  |  |  |
|  | Critical | non-Critical | Total |
| **Disease** | **152** | **47** | 199 |
| **non-Disease** | **183** | **3512** | 3695 |
|  | 335 | 3559 | 3894 |

**Table S3 - Results of the MDS and critical set for ncNRAs from the HMDD and LncRNADisease databases**

This table shows the number of ncRNAs that belong (or not) to an MDS (upper) and critical set (lower) of nodes engaged in network control and are also associated (or not) with more than one disease. Associations between ncRNAs and diseases are obtained from the combined information annotated in the HMDD and LncRNADisease databases.

| **OMiR** |  |  |  |
| --- | --- | --- | --- |
|  | MDS | non-MDS | Total |
| **Disease** | **83** | **85** | 168 |
| **non-Disease** | **288** | **3438** | 3726 |
|  | 371 | 3523 | 3894 |
|  |  |  |  |
|  |  |  |  |
|  | Critical | non-Critical | Total |
| **Disease** | **77** | **91** | 168 |
| **non-Disease** | **258** | **3468** | 3726 |
|  | 335 | 3559 | 3894 |

**Table S4 - Results of the MDS and critical set for ncRNAs from the OMiR database**

This table shows the number of ncRNAs that belong (or not) to an MDS (upper) and critical set (lower) and are also associated (or not) with more than one disease. Associations between ncRNAs and diseases are obtained from the OMiR dataset.
